# Contribution of common risk variants to multiple sclerosis in Orkney and Shetland

**DOI:** 10.1038/s41431-021-00914-w

**Published:** 2021-06-04

**Authors:** Catriona L. K. Barnes, Caroline Hayward, David J. Porteous, Harry Campbell, Peter K. Joshi, James F. Wilson

**Affiliations:** 1 Centre for Global Health Research, Usher Institute, University of Edinburgh, Edinburgh, Scotland; 2 MRC Human Genetics Unit, Institute of Genetics and Molecular Medicine, University of Edinburgh, Edinburgh, Scotland; 3 Centre for Genomic & Experimental Medicine, Institute of Genetics & Molecular Medicine, University of Edinburgh, Edinburgh, UK

**Keywords:** Population genetics, Multiple sclerosis, Genetics research

## Abstract

Orkney and Shetland, the population isolates that make up the Northern Isles of Scotland, are of particular interest to multiple sclerosis (MS) research. While MS prevalence is high in Scotland, Orkney has the highest global prevalence, higher than more northerly Shetland. Many hypotheses for the excess of MS cases in Orkney have been investigated, including vitamin D deficiency and homozygosity: neither was found to cause the high prevalence of MS. It is possible that this excess prevalence may be explained through unique genetics. We used polygenic risk scores (PRS) to look at the contribution of common risk variants to MS. Analyses were conducted using ORCADES (97/2118 cases/controls), VIKING (15/2000 cases/controls) and Generation Scotland (30/8708 cases/controls) data sets. However, no evidence of a difference in MS-associated common variant frequencies was found between the three control populations, aside from HLA-DRB1*15:01 tag SNP rs9271069. This SNP had a significantly higher risk allele frequency in Orkney (0.23, *p* value = 8 × 10^–13^) and Shetland (0.21, *p* value = 2.3 × 10^–6^) than mainland Scotland (0.17). This difference in frequency is estimated to account for 6 (95% CI 3, 8) out of 150 observed excess cases per 100,000 individuals in Shetland and 9 (95% CI 8, 11) of the observed 257 excess cases per 100,000 individuals in Orkney, compared with mainland Scotland. Common variants therefore appear to account for little of the excess burden of MS in the Northern Isles of Scotland.

## Introduction

Multiple sclerosis (MS) is the most common neurological disability found in young adults in the Western world [1]. The disease is characterised by the inflammation and chronic degeneration of the central nervous system, a result of the destruction of the myelin sheath by the individual’s immune system [2]. There is no definitive explanation as to the reason for these immune attacks, with genetic susceptibility and environmental factors both contributing to MS risk [3, 4]. Although environmental factors (for example, vitamin D, smoking, Epstein–Barr viral exposure and body mass index (BMI)) are important for MS risk [5–8], MS has a material genetic component: broad-sense heritability has been estimated using twin, sibling and half-sibling data as 0.64 (with a 95% confidence interval (CI) of 0.36–0.76) [9]. Single-nucleotide polymorphism (SNP) heritability has been estimated at 0.19 (95% CI 0.18, 0.20) [10]. MS arises most frequently in genetically susceptible individuals who may have been exposed to risk-associated environmental factors or stochastic events [11]. A total of 233 genetic variants have been identified as associated with MS susceptibility, including 32 independent genetic effects within the major histocompatibility (MHC) region on chromosome 6 [10]. The majority of these variants have an odds ratio (OR) ranging from 1.05 to 1.20; however, the strongest association identified is with the HLA-DRB1*15:01 variant, with an OR of 2.92 [10, 12].

## MS in the Northern Isles

The highest prevalence of MS in the world is found in the Orkney islands in the North of Scotland, where prevalence reaches 402 individuals per 100,000 [13]. The Shetland islands, 50 miles north of Orkney, have a similarly high rate of 295 individuals per 100,000 [13]. Mainland Scotland, for comparison, has a prevalence of 145 individuals per 100,000 [13]. The prevalence of MS within these island groups, in particular Orkney, is significantly higher than what would be expected for a population of that latitude. A number of previous studies have investigated the potential cause of this excess of MS prevalence. Vitamin D deficiency as a potential cause of MS was investigated by Weiss et al. in 2016; however, plasma 25-hydroxyvitamin D was found to be significantly higher in those on Orkney than in mainland Scotland (mean 35.3 compared to 31.7 nmol/L). Additionally, Orkney had a lower prevalence of severe plasma 25-hydroxyvitamin D deficiency (of 6.6% compared to 16.2% in mainland Scotland) [14]. Another study investigated homozygosity, the inheritance of identical haplotypes from both parents, in Orkney and Shetland [15]. Three measures of genome-wide homozygosity were generated for 88 MS patients and 178 matched controls and assessed for association with MS. However, no association was detected, and so consanguinity is not thought to be the principal cause of the excess MS prevalence. However, it is possible that this excess prevalence may be explained genetically through the Northern Isles having a higher proportion of common risk alleles. If the ancestors or founders of the islands by chance had higher frequencies of these risk alleles, this may cause additional cases of MS as they would be segregating at higher frequencies today. This idea was first suggested in 1981 by Compston, who implied that Orcadians in general may have higher frequencies of common risk variants [16].

## Methods

### Study participants

This research used 97 MS cases and 2118 controls from the Orkney Complex Disease Study and the Northern Isles Multiple Sclerosis Study (NIMS; collectively referred to as ORCADES) as a sample of the Orkney population, 15 MS cases and 2090 controls from the Viking Health Study Shetland (VIKING) as a sample of the Shetland population and 30 MS cases and 8708 controls from Generation Scotland (GS) as a sample of the mainland Scotland population. ORCADES, VIKING and GS are cross-sectional, family-based cohorts that were established to become platform resources for the study of complex disease in Scotland, while the NIMS was established with the specific aim of studying MS. Data collection and genotyping for ORCADES, VIKING and GS has been fully described in previous research papers but has been summarised along with genotype and sample quality control (QC) steps in Supplementary Table 1 [17–19]. A principal component plot of the three cohorts can also be found in Supplementary Fig. 1.

### Selecting common risk variants for polygenic risk score (PRS) calculation

A total of 127 key MS SNPs (Supplementary Table 2) were compiled for PRS calculation, selected from the 2011 International Multiple Sclerosis Genetics Consortium (IMSGC) genome-wide association study (GWAS) [20] and the GWAS Catalogue [21–30]. The SNPs selected from the 2011 IMSGC GWAS comprised a group of 102 SNPs that were taken forward for replication analysis in the original study. The 2011 IMSGC GWAS was chosen in particular as this was the most recent large-scale GWAS that the NIMS cohort did not contribute towards. SNPs were included from the GWAS Catalogue if the disease trait was listed as “multiple sclerosis” and the SNPs originated from studies including only European individuals. Additionally, only studies up to 2012 were considered. Any SNPs originating from the 2011 IMSGC GWAS, or from a study that included any individuals from Orkney, were excluded from the GWAS Catalogue search. Only SNPs with a *p* value <1 × 10^−3^ were considered, and SNPs were only included if they were present in all three cohorts. Strict QC procedures were applied to ensure that PRS results produced from these SNPs were not biased or inaccurate and that subjects from the Northern Isles had not contributed to the underlying GWAS. These QC methods included removing SNPs without a reported OR or risk allele, using inverse variance meta-analyses to determine ORs and *p* values for duplicated SNPs, and clumping SNPs for linkage disequilibrium, using a cut-off threshold of *r*^2^ = 0.25 within a 200-kb window.

### Calculating PRSs

PRSs were calculated for all individuals using the R package PRSice (v1.25), which used the HRC-imputed genotype dosage data for ORCADES, VIKING and GS as the target SNP set, along with SNP effect sizes from the original source (either the GWAS Catalogue or 2011 IMSGC GWAS). Risk scores were produced for the full SNP set (*n* = 127), the SNP set without HLA-DRB1*15:01 tag SNP rs9271069 (*n* = 126) and for rs9271069 alone.

### Differentiating MS cases and controls

PRS were compared between cases and controls in each data set. The three data sets were first standardised by *z*-scoring the PRS to allow comparison between populations. Cases and controls were then compared within each data set by fitting a generalised linear model with Gaussian errors and an identity link function, using the R function *glm*. Age, sex and the first two principal components were included as covariates for all three populations. A meta-analysis for cases and a meta-analysis for controls were performed to determine an estimate for the overall case/control PRS.

### Fitting a logistic regression model

Related individuals were removed before fitting the data to a logistic regression model using a genomic kinship coefficient threshold of 0.05. Following removal of related individuals, cases and control numbers in each data set were as follows: 80/645 ORCADES individuals, 14/642 VIKING individuals, and 29/ 8341 GS individuals. A logistic regression model was fitted separately to each data set using the R function *glm*, with MS disease status as the dependent variable, PRS as the independent variable and age, sex, principal component 1 and principal component 2 included as covariates. A null model (with only covariates) was also fitted to each data set.

### Determining how much MS variance is explained by common risk variants

Logistic regression model results were used to assess how much variance in MS risk common risk variants could explain. Nagelkerke’s pseudo *R*^2^ value was calculated in R for each data set and SNP group (full SNP set, SNP set without rs9271069 and rs9271069 alone) using the model results.

### Determining how successful common risk variants are in predicting MS

To determine whether common risk variants could be a predictor of an individual’s MS status, the model results were used to calculate a receiver operator characteristic curve (ROC) and the area under this curve (AUC). ROCs were plotted for each fitted model to assess how well the PRS with covariates predicted MS disease status. The AUC was also calculated to quantify the predictive ability of each model.

### Comparing common variants between Orkney, Shetland and mainland Scotland

To assess the difference in common risk variants between each population, mean PRS between control individuals were compared between GS, ORCADES and VIKING using two-sample *t* tests. The frequency of each PRS SNP was calculated in individuals without MS and compared between GS, ORCADES and VIKING using a Pearson’s chi-squared test.

### Determining how much risk is explained by common risk variants

An important aspect of this study was to estimate the contribution of these variants to the excess MS prevalence in the Northern Isles. This was done by determining the expected difference in prevalence due to the differences in frequencies of these variants between data sets. The expected difference in prevalence was then compared to the observed difference in prevalence seen in mainland Scotland, Orkney and Shetland. First, the difference in means between (a) GS and VIKING and (b) GS and ORCADES was calculated. Only control individuals were included in this calculation. The differences in means were then multiplied by the beta value produced from the meta-analysis of each of the data set’s models with covariates, to give the expected odds values. The expected odds values produced is the proportion of the odds that could be attributed to difference in mean PRS between populations. Thus, given the frequencies of the common risk variants that have been looked at in the PRS, these values would be the expected increase in MS risk for the Northern Isles populations, using GS (Glasgow/Dundee) as the baseline. This would reflect the genetic difference due to common risk variants. To determine how this expected difference in MS risk explained by common risk variants compared to the observed MS risk between populations, the expected differences in odds values were compared to the observed difference in odds values. Observed MS prevalence data were obtained from Visser et al. [13] using Aberdeen City for Mainland Scotland. ORs were calculated using contingency tables and converted into log of ORs for comparison with the expected values.

## Results

### Data summary

The demographic characteristics for study individuals can be found in Table 1.

**Table 1:** Summary statistics for ORCADES, VIKING and Generation Scotland.

Population	Sex	Count			Mean age (standard deviation)		
		Case	Control	Total	Case	Control	Total
ORCADES	M	28	843	871	54.30 (9.40)	54.85 (15.20)	54.83 (15.04)
	F	69	1275	1344	49.13 (12.36)	53.85 (15.54)	53.61 (15.43)
	All	97	2118	2215	50.64 (11.76)	54.25 (15.41)	54.09 (15.28)
VIKING	M	3	839	842	60.95 (8.83)	51.34 (15.47)	51.37 (15.46)
	F	12	1251	1263	53.73 (13.10)	48.93 (15.06)	48.97 (15.05)
	All	15	2090	2105	55.28 (12.39)	49.90 (15.27)	49.93 (15.26)
Generation Scotland	M	5	3574	3579	45.80 (7.92)	45.89 (15.26)	45.89 (15.26)
	F	25	5134	5159	50.80 (9.53)	46.48 (14.78)	46.50 (14.76)
	All	30	8708	8738	49.97 (9.35)	46.23 (14.98)	46.25 (14.97)

Count and mean age for the Orkney Complex Disease Study (ORCADES), Viking Health Study – Shetland (VIKING) and Generation Scotland cohorts, split by gender and multiple sclerosis (MS) disease status.

### Comparing PRS in MS cases and controls

**Fig. 1:**
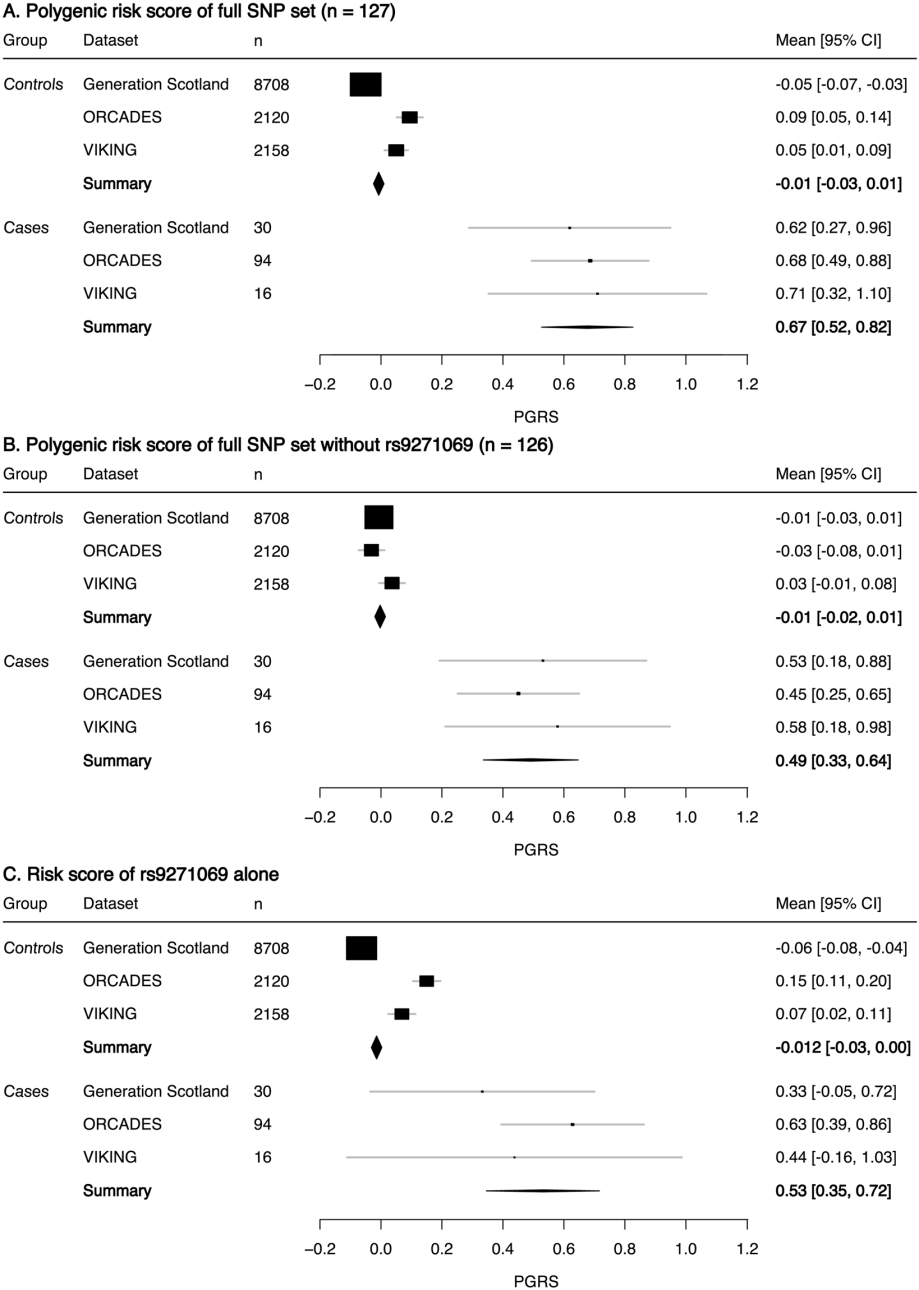
Forest plots of *z*-scored polygenic risk scores (PRSs) for multiple sclerosis cases and controls in Generation Scotland, ORCADES and VIKING. PRS calculated using three SNP sets are used for comparison: A. the full SNP set (*n* = 127), B. the SNP set without HLA-DRB1*15:01 tag SNP rs9271069 (*n* = 126), and C. a risk score for rs9271069 alone.

MS cases have statistically significantly higher polygenic scores than MS controls using the full SNP set in each population (Fig. 1 and Supplementary Table 3, with *p* values: ORCADES, 5.63 × 10^–9^; GS, 7.6 × 10^–4^; VIKING, 2.82 × 10^–2^), validating the use of PRS. When rs9271069 is removed, only cases in ORCADES (*p* value = 1.8 × 10^–5^) and GS (*p* value = 5.67 × 10^–3^) are statistically different. When rs9271069 is considered alone, only ORCADES cases and controls are statistically different (*p* value = 1.02 × 10^–5^), with slight significance seen in GS (*p* value = 0.045). This pattern of significance reflects the number of cases found in each data set: ORCADES (significant in all three comparisons) has 97 cases, GS (significant in two comparisons) has 30 cases, and VIKING (significant in the main comparison only) has 15 cases.

### How much variance PRS explain and their ability to predict MS

To determine how much MS variance is explained by common risk variants, a logistic regression model was fitted separately to each data set, with MS status as the dependent variable, PRS as the independent variable, and age, sex, PC 1 and PC2 as covariates. Nagelkerke’s pseudo *R*^2^ and AUC values were calculated using the model results to determine how much variance the risk scores explained and how successful they were in predicting MS.

The full model results, including *R*^2^ and AUC values, can be found in Table 2. The models fitted using the full SNP set (*n* = 127) with covariates resulted in the highest *R*^2^ values in each population (VIKING *R*^2^ = 0.075, ORCADES *R*^2^ = 0.070, GS *R*^2^ = 0.069). In comparison, the null model (containing only covariates) for each population resulted in an *R*^2^ value of 0.045 for VIKING, 0.019 for ORCADES and 0.038 for GS.

**Table 2:** Logistic regression results for predicting MS risk using polygenic risk scores.

Model	Estimate	SE	*t* value	Pr(>|*t*|)	Sig.	AIC	*R*^2^	AUC	AUC SE
VIKING: Full SNP set	0.59	0.26	2.26	0.024	*	167.73	0.075	0.762	0.055
VIKING: HLA only	0.31	0.23	1.37	0.172		170.99	0.055	0.706	0.066
VIKING: SNP set without HLA	0.51	0.27	1.86	0.063		169.27	0.066	0.753	0.047
VIKING: Null model						170.72	0.045	0.696	0.056
ORCADES: Full SNP set	0.60	0.11	5.58	2.36 × 10^–8^	***	696.36	0.070	0.705	0.029
ORCADES: HLA only	0.40	0.09	4.31	1.60 × 10^–5^	***	710.31	0.047	0.658	0.032
ORCADES: SNP set without HLA	0.45	0.11	4.11	3.98 × 10^–5^	***	710.85	0.046	0.666	0.030
ORCADES: Null model						725.93	0.019	0.600	0.029
Generation Scotland: Full SNP set	0.63	0.19	3.40	0.001	***	372.32	0.069	0.765	0.042
Generation Scotland: HLA only	0.32	0.16	1.96	0.050		380.24	0.048	0.731	0.045
Generation Scotland: SNP set without HLA	0.53	0.19	2.81	0.005	**	375.83	0.059	0.743	0.045
Generation Scotland: Null model						381.73	0.038	0.714	0.052

Logistic regression results for predicting MS risk using PRS, using ORCADES, VIKING and Generation Scotland cohorts. MS status was used as the dependent variable in each model. Age, sex, principal component 1 and principal component 2 were used as covariates for all models. Three separate PRS were used as the independent variable within each population: the full SNP set (*n* = 127 SNPs), the full SNP set without HLA-DRB1*15:01 SNP rs9271069 (*n* = 126), and for the HLA-DRB1*15:01 SNP rs9271069 alone (*n* = 1). The null model was fitted without any PRS. AIC values, Nagelkerke’s *R*^2^ values and AUC values (with associated standard error values) are also included for each model. For ease of readability, only the coefficients of the genetic effect are shown. Thus, the coefficients of the covariates are not included. For the null models, only the AIC, Nagelkerke’s *R*^2^ and AUC values are shown, as there is no genetic effect included within the null model. The Sig. column denotes the signficance of the model estimate, where *** indicates a *p* value < 0.001, ** indicates a *p* value < 0.01 and * indicates a *p* value < 0.05.

The models fitted using the full SNP set (*n* = 127) with covariates also resulted in the highest AUC values for each population: GS AUC = 0.77 (95% CI 0.68–0.85), VIKING AUC = 0.76 (0.65–0.87) and ORCADES AUC = 0.70 (0.65–0.76). In comparison, the null model (containing only covariates) for each population resulted in AUC values of 0.71 (0.61–0.81) for GS, 0.70 (0.59–0.81) for VIKING and 0.60 (0.54–0.66) for ORCADES. The ROC curves for each population and PRS can be found in Supplementary Fig. 1.

### Do MS common risk variants differ in frequency between mainland Scotland and the Northern Isles?

When we compared the control populations from each data set, a statistically significant difference between GS and both ORCADES and VIKING was seen for the full SNP set (Table 3). When rs9271069 was removed from the SNP set, there was no significant difference detectable between any of the control populations. When rs9271069 was looked at alone, a statistical difference was seen when comparing all populations. Supplementary Table 2 provides the risk allele frequency (RAF) of each PRS SNP, calculated using control individuals within each population, along with Pearson’s chi-squared results comparing RAF between populations. The SNP with the highest associated MS risk, HLA-DRB1*15:01 tag SNP rs9271069 (OR = 2.77), had a significantly higher frequency in Orkney controls (RAF = 0.23) and Shetland controls (RAF = 0.21) than mainland Scotland controls (RAF = 0.17: respective *p* values of 8 × 10^–13^ and 2.3 × 10^–6^).

**Table 3:** Comparison of PRS of MS controls between populations.

SNP set	Population	Controls (*n*)	Mean PRS	Population	Controls (*n*)	Mean PRS	*t* test statistic	*p* value
Full	GS	8708	−0.047	ORCADES	2120	0.094	−5.78	8.34 × 10^–9^
	GS	8708	−0.047	VIKING	2158	0.052	−4.05	5.26 × 10^–5^
	ORCADES	2120	0.094	VIKING	2158	0.052	1.35	0.18
Without rs9271069	GS	8708	−0.01	ORCADES	2120	−0.033	1.03	0.3
	GS	8708	−0.01	VIKING	2158	0.034	−1.77	0.08
	ORCADES	2120	−0.033	VIKING	2158	0.034	−2.22	0.03
rs9271069 only	GS	8708	−0.063	ORCADES	2120	0.151	−8.54	2.11 × 10^–17^
	GS	8708	−0.063	VIKING	2158	0.07	−5.33	1.05 × 10^–7^
	ORCADES	2120	0.151	VIKING	2158	0.07	2.53	0.01

Two-sided *t* test results comparing *z*-score transformed polygenic risk scores (PRS) of multiple sclerosis controls between Generation Scotland, ORCADES and VIKING, using PRS produced using the full SNP set (*n* = 127), the SNP set without HLA-DRB1*15:01 tag SNP rs9271069 (*n* = 126) and a risk score for rs9271069 alone.

### How much of the excess MS cases in the Northern Isles is caused by common risk variants?

To determine the contribution of common risk variants to excess MS cases in the Northern Isles, a comparison was made between the calculated expected odds and the observed odds seen from MS prevalence data (Table 4). In Shetland, common risk variants account for 9 (95% CI 5, 14) out of 150 observed excess cases per 100,000 individuals. The majority of the expected odds is from the HLA-DRB1*15:01 tag SNP rs9271069, which contributes an equivalent of 6 cases (95% CI 3, 8) per 100,000 individuals. In Orkney, all the expected excess genetic odds are due to HLA-DRB1*15:01 tag SNP rs9271069, which accounts for 9 cases (95% CI 8, 11) of the observed 257 excess cases per 100,000 individuals. Considering the populations of Orkney (22,000) and Shetland (23,000), the differences in allele frequencies of common variants can thus only explain the equivalent of about two excess cases of MS in each archipelago.

**Table 4:** The contribution of common risk variants to excess MS prevalence in the Northern Isles.

		Generation Scotland	VIKING (95% CI)	ORCADES (95% CI)
Expected excess MS risk due to all common risk variants	Log(OR)	0	**0.06 (0.03, 0.09)**	**0.05 (0.02, 0.08)**
	Equivalent number of cases	0	9 (5, 14)	8 (5, 11)
Expected excess MS risk due to common risk variants without HLA-DRB1*1501 SNP rs9271069	Log(OR)	0	**0.02 (0.00, 0.05)**	**−0.01 (−0.04, 0.01)**
	Equivalent number of cases	0	3 (0, 6)	−1 (−3, 2)
Expected excess MS risk due to HLA-DRB1*1501 SNP rs9271069	Log(OR)	0	**0.04 (0.02, 0.05)**	**0.06 (0.05, 0.07)**
	Equivalent number of cases	0	6 (3, 8)	9 (8, 11)
Observed excess MS risk in populations	Log(OR)	0	0.71 (0.51, 0.91)	1.02 (0.83, 1.21)
	Equivalent number of cases	0	150^a^	257^a^

Expected and observed excess MS risk (log of odds ratios) in both VIKING (Shetland; *n* controls = 2158) and ORCADES (Orkney; *n* controls = 2120) when compared to Generation Scotland (*n* controls = 8708). The difference between Generation Scotland and either ORCADES or VIKING for expected MS risk differences are highlighted in bold. Expected log(OR) values were calculated from the logistic regression results by multiplying the coefficient from the model with the mean PRS calculated from the full SNP set (*n* = 127), the full SNP set without HLA-DRB1*15:01 SNP rs9271069 (*n* = 126) and HLA-DRB1*15:01 SNP rs9271069 alone (*n* = 1). Observed log of odds values were calculated from the prevalence data found in the paper by Visser et al. The logistic regression model was fit to the cohort PRS data, using MS as the dependent variable, PRS as the independent variable and age, sex and the first two principal components.

^a^Taken directly from the observed data.

### Study limitations

A major limitation of this study was the use of the IMSGC 2011 GWAS as a key contributor to the SNP data on which the PRS calculation was based. It was preferable to use the most recent, large-scale GWAS for this study; however, the IMSGC 2011 GWAS is the most recent GWAS that did not include data generated from individuals in the NIMS. While using an older GWAS may result in some loss of power, we have included the most important common susceptibility alleles within the PRS calculation in this study. We also recognise that this PRS uses a limited number of strong-effect SNPs selected from multiple sources; while this does not bias our results, there are many ways to construct a PRS and this score could have been made in other ways by other researchers at other times.

The sample size was a further limitation to this study. Orkney and Shetland are both relatively small populations (approximately 22,000 individuals in Orkney and 23,000 individuals in Shetland) and will therefore yield small numbers of MS cases, despite the high rates of MS in the islands. However, the key analyses within this study were to determine: (i) whether common risk variants differed between the Northern Isles and mainland Scotland and (ii) how these variants, as a group, contribute to the excess burden of MS. Both of these analyses were conducted using only control individuals and so were not affected by the small number of cases.

Orkney and Shetland remain of key interest to MS: while it is not possible to increase the case numbers within ORCADES and VIKING, it was important to utilise these cohorts given the unique genetics of these population isolates.

### Future work

The PRS used in this study included the strongest effect allele from the MHC region, HLA-DRB1*15:01. The MHC region is complex and includes other high-risk variants (which may be in linkage disequilibrium with the SNP included in this study). It is suggested that a future study using PRS for MS focus on the MHC region specifically.

## Discussion

We found that common risk variants do not make a material contribution towards the higher rates of MS in the Northern Isles of Scotland. However, a small proportion of excess risk can be attributed to a tag SNP for HLA-DRB1*15:01, the major single genetic risk factor for MS (OR = 2.92). This SNP has a significantly higher frequency in the Northern Isles, particularly Orkney (RAF = 0.23), than mainland Scotland (RAF = 0.17, *p* value = 8 × 10^–13^). Frequencies for the tag SNP for HLA-DRB1*15:01 reported in other cohorts appears to be similar to that in mainland Scotland or lower: the ALFA Allele Frequency project reports the frequency within European populations as 0.14 (sample size 33,120) [31]. It is possible that the frequency of this allele is higher in the Northern Isles populations due to the founders of Orkney and Shetland having higher frequencies of the risk allele or it may have risen through genetic drift in the past thousand years. Regardless, the significantly higher frequency of this SNP in the Northern Isles in comparison to both mainland Scotland and the general European population suggests that it plays a modest role in the prevalence of MS in the Northern Isles, even if not the excess prevalence. We found that PRSs calculated with the 127 most strongly associated MS risk variants explained approximately 3–5% of variance. The 2019 IMSGC study, which analysed data from 47,429 MS cases and 68,374 control subjects, estimated the heritability attributable to all analysed common genetic variants at 19.2% [10]. Of this variance, 18% was explained by genome-wide significant variants. Therefore, the heritability of genome-wide significant variants was estimated at approximately 3.5%, which aligns with the estimate from this study. Additionally, the variance within this study is largely dominated by the increased frequency of the HLA-DRB1*15:01 SNP in ORCADES, offsetting any gain from including the additional SNPs with small (and uncertain) effects. The predictive capacity of the PRSs for MS status was calculated with an AUC of 0.77 (95% CI 0.68–0.85) in GS, 0.76 (0.65–0.87) in VIKING and 0.70 (0.65–0.76) in ORCADES. This is in line with the scores previously published in literature. A 2016 study using 452 MS cases with 103 common risk variants estimated the AUC to be 0.72 (95% CI 0.69, 0.75), which overlaps with the results produced here [32]. MS is a heterogenous disease, and while it is useful to see to what degree genetic risk scores could aid in prediction, additional, especially rare, variants or the incorporation of environmental factors into prediction models will be required to improve risk prediction. The cause of the excess MS prevalence in the Northern Isles remains mostly unexplained and is still a case for investigation: it can likely be attributed to several factors. One hypothesis is that a number of rare susceptibility variants of large effect are segregating in the islands. Much evidence has been presented in recent years to support the influence of rare variants in MS risk, particularly among family groups [33]. Within the Northern Isles, it is possible that there are one or more rare variants, drifted to higher frequency due to genetic simplification, possibly segregating within families, that are associated with MS. Smaller contributions will also be made from the high rates of overweight and obesity found in the islands. Orkney has the highest percentage of individuals who are classed as overweight and obese for any location in Scotland at 73% (95% CI 68, 78), while Shetland stands at 71% (95% CI 64, 77). For comparison, the Scottish average is 65% (95% CI 64, 66) [34]. Obesity is a known risk factor for MS; a Mendelian Randomisation study showed that an increase of 1 standard deviation in BMI increased the odds of developing MS by 41% (95% CI 20, 66) [8]. Additionally, interactive effects have been shown between HLA-DRB1*15:01 and obesity: individuals classed as obese who carry HLA-DRB1*15 but not HLA-A*02 have an OR of 16.2 (95% CI 7.5, 35.2) for developing MS [35]. Given that there are most likely multiple contributors to the excess rate of MS in the Northern Isles, there are numerous avenues for future research. The most obvious of these appears to be a targeted search for rare variants. Whole-genome sequence analysis has detected many Shetland-specific rare variants [36]. The similar strong differentiation of Orkney in genome-wide population genetic analyses [37] predicts a similar situation there; however, power will always be limited by the low absolute numbers of cases, overall and in any particular kindred. The future of MS research in the Northern Isles and beyond will lie in new methods to discover and explore the function of susceptibility variants and their interactions with their unique environment. GWAS summary statistics used to create the PRS and the raw PRS for each study population can be found within the Edinburgh DataShare (10.7488/ds/2992).

## Supplementary information

Supplementary Figure Legends

Supplementary Figure 1

Supplementary Figure 2

Supplementary Tables
