# Supplementary material for: Contribution of common risk variants to multiple sclerosis in Orkney and Shetland
Source: Eur J Hum Genet. 2021 Jun 4;29(11):1701–9. doi: 10.1038/s41431-021-00914-w (PMC8560837; doi:10.1038/s41431-021-00914-w)
Supplement: Supplementary file 1 — Supplementary Figure Legends [file 41431_2021_914_MOESM1_ESM.docx]

**Supplementary Figure Legends**

Supplementary Figure 1: Principal component plot for VIKING, ORCADES and Generation Scotland cohorts, using the first two principal components. Multiple sclerosis cases and controls are plotted in separate colours.

Supplementary Figure 2: ROC curves for each population showing the average predictive of predicting multiple sclerosis status using PRS, with covariates age, sex, PC1 and PC2. PRS calculated using three separate SNP sets are used: the full SNP set (n = 127), the SNP set without *HLA-DRB1*15:01* tag SNP rs9271069 (n = 126) and *HLA-DRB1*15:01* tag SNP rs9271069 alone (n = 1).
