# Supplementary figures and images for: Contribution of common risk variants to multiple sclerosis in Orkney and Shetland
Source: Eur J Hum Genet. 2021 Jun 4;29(11):1701–9. doi: 10.1038/s41431-021-00914-w (PMC8560837; doi:10.1038/s41431-021-00914-w)

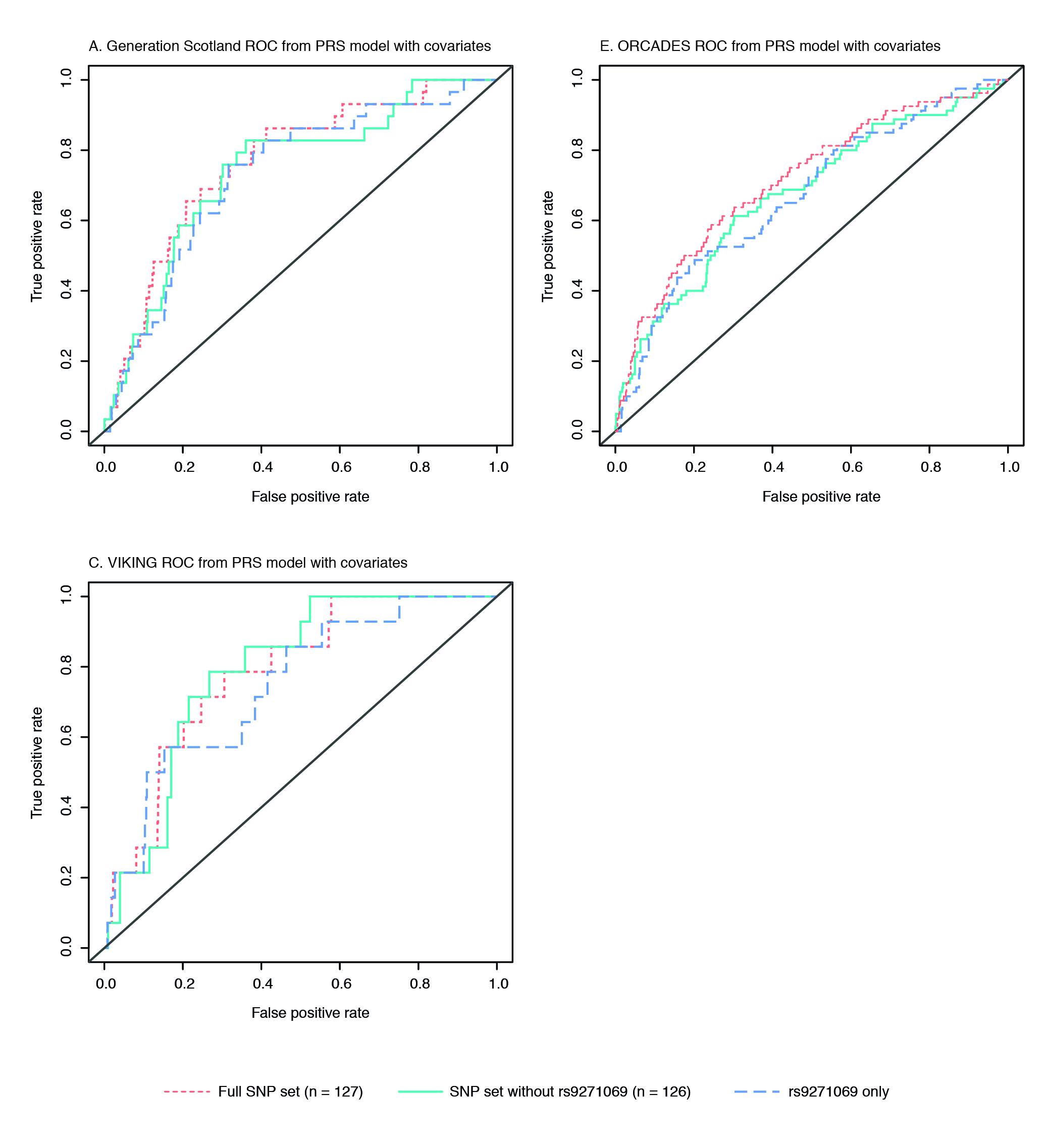

Supplement: Supplementary file 3 — Supplementary Figure 2 [file 41431_2021_914_MOESM3_ESM.jpg]
